# δ‐Tocotrienol sensitizes and re‐sensitizes ovarian cancer cells to cisplatin via induction of G1 phase cell cycle arrest and ROS/MAPK‐mediated apoptosis

**DOI:** 10.1111/cpr.13111

**Published:** 2021-09-14

**Authors:** Fabrizio Fontana, Monica Marzagalli, Michela Raimondi, Valentina Zuco, Nadia Zaffaroni, Patrizia Limonta

**Affiliations:** ^1^ Department of Pharmacological and Biomolecular Sciences Università degli Studi di Milano Milan Italy; ^2^ Department of Applied Research and Technological Development Molecular Pharmacology Unit Fondazione IRCCS Istituto Nazionale dei Tumori Milan Italy

**Keywords:** apoptosis, cisplatin, MAPK, ovarian cancer, ROS, tocotrienols

## Abstract

**Objectives:**

Among gynaecologic malignancies, ovarian cancer (OC) represents the leading cause of death for women worldwide. Current OC treatment involves cytoreductive surgery followed by platinum‐based chemotherapy, which is associated with severe side effects and development of drug resistance. Therefore, new therapeutic strategies are urgently needed. Herein, we evaluated the anti‐tumour effects of Vitamin E‐derived δ‐tocotrienol (δ‐TT) in two human OC cell lines, IGROV‐1 and SKOV‐3 cells.

**Materials and Methods:**

MTT and Trypan blue exclusion assays were used to assess δ‐TT cytotoxicity, alone or in combination with other molecules. δ‐TT effects on cell cycle, apoptosis, ROS generation and MAPK phosphorylation were investigated by flow cytometry, Western blot and immunofluorescence analyses. The synergism between δ‐TT and chemotherapy was evaluated by isobologram analysis.

**Results:**

We demonstrated that δ‐TT could induce cell cycle block at G1‐S phase and mitochondrial apoptosis in OC cell lines. In particular, we found that the proapoptotic activity of δ‐TT correlated with mitochondrial ROS production and subsequent JNK and p38 activation. Finally, we observed that the compound was able to synergize with cisplatin, not only enhancing its cytotoxicity in IGROV‐1 and SKOV‐3 cells but also re‐sensitizing IGROV‐1/Pt1 cell line to its anti‐tumour effects.

**Conclusions:**

δ‐TT triggers G1 phase cell cycle arrest and ROS/MAPK‐mediated apoptosis in OC cells and sensitizes them to platinum treatment, thus representing an interesting option for novel chemopreventive/therapeutic strategies for OC.

## Graphical abstract

δ‐TT triggers G1 phase cell cycle arrest and ROS/MAPK‐mediated apoptosis in OC cells, sensitizing and re‐sensitizing them to platinum treatment.

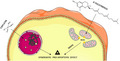

## 1 INTRODUCTION

Ovarian cancer (OC) is the seventh most commonly diagnosed cancer among women worldwide, representing the deadliest form of gynaecologic malignancy.^1^ Due to the lack of early symptoms and consequent difficulties in detection, most OC patients are diagnosed at advanced stages.^2^, ^3^ Current standard treatment for patients with advanced OC includes primary tumour cytoreductive surgery followed by chemotherapy.^4^

Platinum is commonly used in OC chemotherapy.^4^ The efficacy of this DNA‐damaging agent seems to be correlated with its ability to inhibit the proliferation and induce apoptosis of cancer cells. However, despite being initially responsive, about 70% of patients experience tumour relapse a few months after treatment and eventually develop resistance to therapy. Chemotherapy resistance, whether intrinsic or acquired, represents the major challenge in OC management.^5^, ^6^ Additionally, platinum‐based chemotherapy is associated with multiple severe side effects, including nausea, vomiting, myelosuppression, neurotoxicity, nephrotoxicity, hepatotoxicity and ototoxicity.^7^ For all these reasons, more effective and better‐tolerated therapeutic options are urgently needed.

Tocotrienols (TTs) are Vitamin E derivatives endowed with several anti‐tumour properties.^8^, ^9^ We previously demonstrated that δ‐TT could induce ROS‐mediated cell death in prostate cancer and melanoma.^10^, ^11^, ^12^ Moreover, δ‐TT has been recently reported to synergize with bevacizumab in a phase II trial conducted on chemotherapy‐refractory OC.^13^ However, the molecular mechanisms underlying δ‐TT anti‐tumour activity in OC cells have not been elucidated yet.

The present study is aimed at exploring the antiproliferative and proapoptotic effects of δ‐TT on human OC cells, with a focus on its pro‐oxidant action and on its synergistic combination with cisplatin.

## 2 MATERIALS AND METHODS

### 2.1 Chemicals

δ‐TT (average final purity >98%) was isolated from a commercial extract of Annatto seeds (Bixa Orellana L.) (kindly provided by American River Nutrition Inc), as previously described.^14^ Cisplatin was from Sigma‐Aldrich.

The following primary antibodies were utilized: cyclin D1 (2978), cyclin D3 (2936), CDK4 (12790), CDK6 (3136), p21 (2947), p27 (3686), Bax (2772), Bcl‐2 (4223), caspase 9 (9502), caspase 3 (9656), cleaved caspase 3 (9664), PARP (9542), JNK (9252), p‐JNK (4668), p38 (8690), p‐p38 (4511) (all from Cell Signaling Technology Inc), cytochrome *c* (sc‐13560) (Santa Cruz Biotechnology Inc) and α‐tubulin (T6199) (Sigma‐Aldrich). All the antibodies were used at the concentration 1:1000, except for cleaved caspase 3 (1:500).

Horseradish‐peroxidase‐conjugated secondary antibody and enhanced chemiluminescence reagents were from Cyanagen Srl.

Alexa Fluor 488 secondary antibody was from Thermo Fisher Scientific.

The pan‐caspase inhibitor Z‐VAD‐FMK (FMK001) was from R&D System Inc. The ROS scavenger NAC (N‐acetyl‐L‐cysteine) as well as JNK (SP600125) and p38 (SB203580) inhibitors were from Sigma‐Aldrich.

### 2.2 Cell lines and cell culture

Cisplatin‐sensitive (IGROV‐1) and cisplatin‐resistant (SKOV‐3) human OC cells were from American Type Culture Collection (ATCC), while IGROV‐1/Pt1 cell line, exhibiting a stable drug‐tolerant phenotype, was obtained by continuous exposure of parental cells to platinum‐based chemotherapy.^15^ They were all cultured in RPMI medium supplemented with 10% FBS, glutamine and antibiotics, in humidified atmosphere of 5% CO_2_/95% air at 37°C. Original stocks of cells were stored frozen in liquid nitrogen; after resuscitation, cells were kept in culture for no more than 10‐12 weeks. Cells were detached through trypsin‐EDTA solution and passaged once/week.

### 2.3 MTT viability assay

Cells were seeded at a density of 3 × 10^4^ cells/well in 24‐well plates for 24 hours and then exposed to the specific compounds. The medium was then changed with MTT solution (0.5 mg/ml) in RPMI without phenol red and FBS; cells were incubated at 37°C for 30 minutes, and violet precipitate was dissolved with isopropanol. Absorbance at 550 nm was measured through an EnSpire Multimode Plate reader (PerkinElmer).

### 2.4 Trypan blue exclusion assay

Cells were plated (1.5 × 10^5^ cells/dish) in 6 cm dishes. After 24 hours, cells were treated with δ‐TT (5‐20 μg/ml, 24 hours). Adherent (viable) and floating (dead) cells were harvested, stained with Trypan blue 0.4% (1:1 v/v) and counted by Luna automated cell counter (Logos Biosystems).

### 2.5 Cell cycle analysis

Cells were plated (1.5 × 10^5^ cells/dish) in 6 cm dishes. After 24 hours, cells were treated with δ‐TT (15 μg/ml, 24 hours). Adherent (viable) and floating (dead) cells were harvested, washed in PBS, fixed in methanol and resuspended in Invitrogen™ FxCycle™ PI/RNase Staining Solution, according to the manufacturer's protocol. The flow cytometry analyses were performed with a Novocyte3000 instrument (ACEA Biosciences). Data were analysed with Novoexpress software.

### 2.6 Annexin V/PI apoptosis assay

Cells were plated (1.5 × 10^5^ cells/dish) in 6 cm dishes. After 24 hours, cells were treated with δ‐TT (15 μg/ml, 24 hours). Adherent (viable) and floating (dead) cells were harvested, washed in PBS and incubated with Annexin V and PI, using the eBioscience™ Annexin V‐FITC Apoptosis Detection Kit. The flow cytometry analyses were performed with a Novocyte3000 instrument (ACEA Biosciences). Data were analysed with Novoexpress software.

### 2.7 Measurement of mitochondrial membrane potential (ΔΨm)

Cells were plated (1.5 × 10^5^ cells/dish) in 6‐cm dishes. After 24 hours, cells were treated with δ‐TT (15 μg/mL, 12 hours). Adherent (viable) and floating (dead) cells were harvested, washed in PBS and incubated with MitoTracker Orange CMTMRos (Thermo Fisher Scientific) 10 nmol/L for 30 minutes. The flow cytometry analyses were performed with a Novocyte3000 instrument (ACEA Biosciences). Data were analysed with Novoexpress software.

### 2.8 Measurement of ROS production

Cells were plated (1.5 × 10^5^ cells/dish) in 6 cm dishes. After 24 hours, cells were treated with δ‐TT (15 μg/ml, 12 hours). Adherent (viable) and floating (dead) cells were harvested, washed in PBS and incubated with 2',7'‐dichlorofluorescin diacetate (Sigma‐Aldrich) 10 μmol/L for 45 minutes or MitoSOX Red (Thermo Fisher Scientific) 5 μmol/L for 10 minutes, to assess overall cellular and mitochondrial ROS levels, respectively. The flow cytometry analyses were performed with a Novocyte3000 instrument (ACEA Biosciences). Data were analysed with Novoexpress software.

### 2.9 Detection of mitochondrial cytochrome *c* release

Cells were seeded at 3 × 10^4^ cells/well in 24‐well plates on polylysine‐coated 13 mm coverslips for 48 hours and then treated with δ‐TT. After 12 hours of treatment, cells were fixed and stained with the specific primary and secondary antibody. Labelled cells were examined under a Zeiss Axiovert 200 microscope with a 63 × 1.4 objective lens linked to a Coolsnap Es CCD camera (Roper Scientific‐Crisel Instruments). Cytochrome *c* release was quantified as the percentage of cells with no cytochrome *c*/mitochondria co‐localization relative to the total number of cells; at least 200 randomly selected cells in multiple fields were assessed.

To confirm the above data, cytochrome *c* release was also evaluated by Western blot analysis. In particular, the cytosolic fraction was separated by the overall cellular protein extract by using a Cell Fractionation Kit (Abcam), and cytochrome *c* expression was analysed in both total cellular and cytoplasmic compartments.

### 2.10 Western blot analysis

Cells were seeded at 5 × 10^5^ cells/dish in 10 cm dishes. After each treatment, cells were lysed in RIPA buffer; protein preparations (25 μg) were resolved on SDS‐PAGE and transferred to nitrocellulose membranes. Membranes were incubated with the specific primary antibodies. Detection was done using horseradish peroxidase‐conjugated secondary antibodies and enhanced chemiluminescence (Westar Eta C Ultra 2.0). α‐Tubulin was utilized as a loading control.

### 2.11 Isobologram analysis

Cells were treated for 24 hours using six different concentrations of cisplatin and δ‐TT 15 μg/ml. Viable cells were quantitated by MTT assay as described above, and CalcuSyn software was used to generate an isobologram.

### 2.12 Statistical analysis

Statistical analysis was performed with a statistic package (GraphPad Prism5, GraphPad Software). Data are represented as the mean ± SEM of three‐four independent experiments. Differences between groups were assessed by t test or one‐way analysis of variance (ANOVA) followed by Dunnett's or Bonferroni's test. A *P* value <0.05 was considered statistically significant.

## 3 RESULTS

### 3.1 δ‐TT reduces OC cell viability and proliferation

**FIGURE 1:**
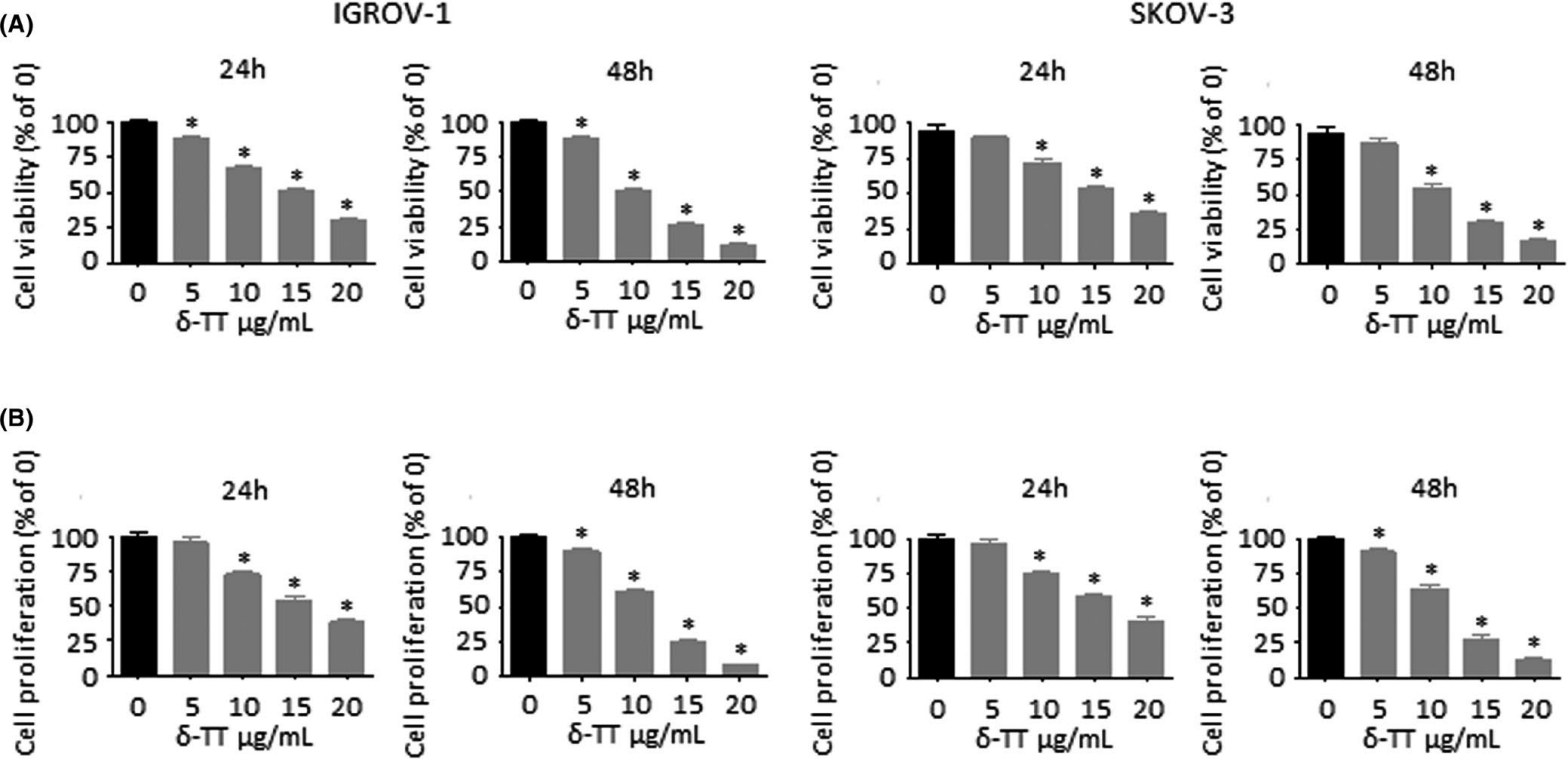
δ‐TT reduces OC cell viability and proliferation. (A), IGROV‐1 and SKOV‐3 cells were treated with δ‐TT (5‐20 μg/ml) for 24 and 48 h. Cell viability was then evaluated by MTT assay. Each experiment was repeated three times. Data represent mean values ± SEM and were analysed by Dunnett's test after one‐way analysis of variance. *P < .05 vs C, controls (vehicle). (B), Cells were treated with δ‐TT (5‐20 μg/ml) for 24 and 48 h. Cell proliferation was then evaluated by Trypan blue exclusion assay. Each experiment was repeated three times. Data represent mean values ± SEM and were analysed by Dunnett's test after one‐way analysis of variance. *P < .05 vs (C), controls (vehicle)

IGROV‐1 and SKOV‐3 cells were treated with crescent doses of δ‐TT (5‐20 μg/ml) for 24 and 48 hours, and cell viability was measured by MTT assay. δ‐TT decreased the number of viable OC cells in a dose‐ and time‐dependent manner (Figure 1A), with IC50 values at 24 hours of 3.60 × 10^−5^ M and 3.90 × 10^−5^ M for IGROV‐1 and SKOV‐3 cells, respectively. The same treatment also dose and time dependently reduced the proliferation of OC cells, as evidenced by Trypan blue exclusion assay (Figure 1B). Based on these observations, we selected δ‐TT 15 μg/ml, a cytotoxic dose close to its IC50, as appropriate concentration for further analyses. Notably, this dosage is consistent with that utilized in various cancer cell lines, as well as comparable to the plasma concentration observed in pancreatic cancer patients recruited for a phase I pharmacokinetic study conducted by Springett and colleagues.^16^, ^17^, ^18^, ^19^, ^20^, ^21^

### 3.2 δ‐TT promotes G1 phase cell cycle arrest in OC cells

**FIGURE 2:**
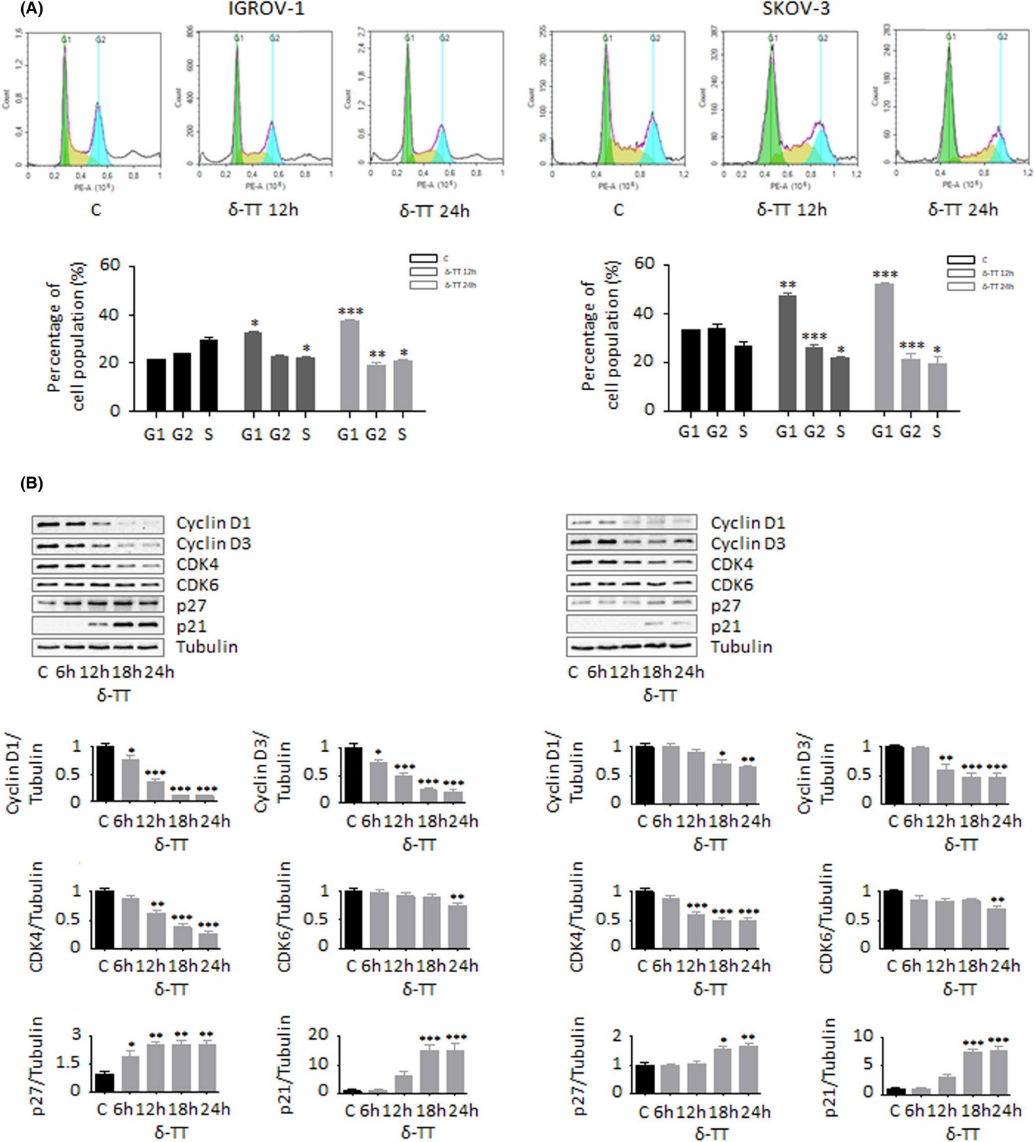
δ‐TT promotes G1 phase cell cycle arrest in OC cells. (A), IGROV‐1 and SKOV‐3 cells were treated with δ‐TT (15 μg/ml, 1‐24 h); cell cycle distribution was then evaluated by cytofluorimetric analysis after staining with Invitrogen™ FxCycle™ PI/RNase Staining Solution (according to the manufacturer's protocol). Each experiment was repeated three times. Data represent mean values ± SEM and were analysed by *t* test. *P < .05 vs C, controls (vehicle). **P < .01 vs C, controls (vehicle). ***P < .001 vs C, controls (vehicle). (B), After δ‐TT treatment (15 μg/ml, 1‐24 h), Western blot analysis was performed to investigate the expression levels of cyclin D1, cyclin D3, CDK4, CDK6, p21 and p27. Tubulin expression was evaluated as a loading control. One representative of three experiments performed is shown. Data represent mean values ± SEM and were analysed by Dunnett's test after one‐way analysis of variance. *P < .05 vs C, controls (vehicle); **P < .01 vs C, controls (vehicle); ***P < .001 vs C, controls (vehicle)

To determine how δ‐TT treatment could affect OC cell growth, cell cycle distribution was assessed by flow cytometry. Our results show that the compound (15 μg/ml, 24 hours) significantly increased the number of both IGROV‐1 and SKOV‐3 cells in G1 phase as compared to control group (Figure 2A). Supporting these results, the expression levels of cell cycle regulation proteins involved in G1/S transition were evaluated by Western blot. As evidenced in Figure 2B, the protein levels of cell cycle promoters cyclin D1, cyclin D3, CDK4 and CDK6 were time‐dependently reduced after δ‐TT treatment, while the expression of cell cycle inhibitors p21 and p27 was parallelly enhanced.

### 3.3 δ‐TT triggers mitochondrial apoptosis in OC cells

**FIGURE 3:**
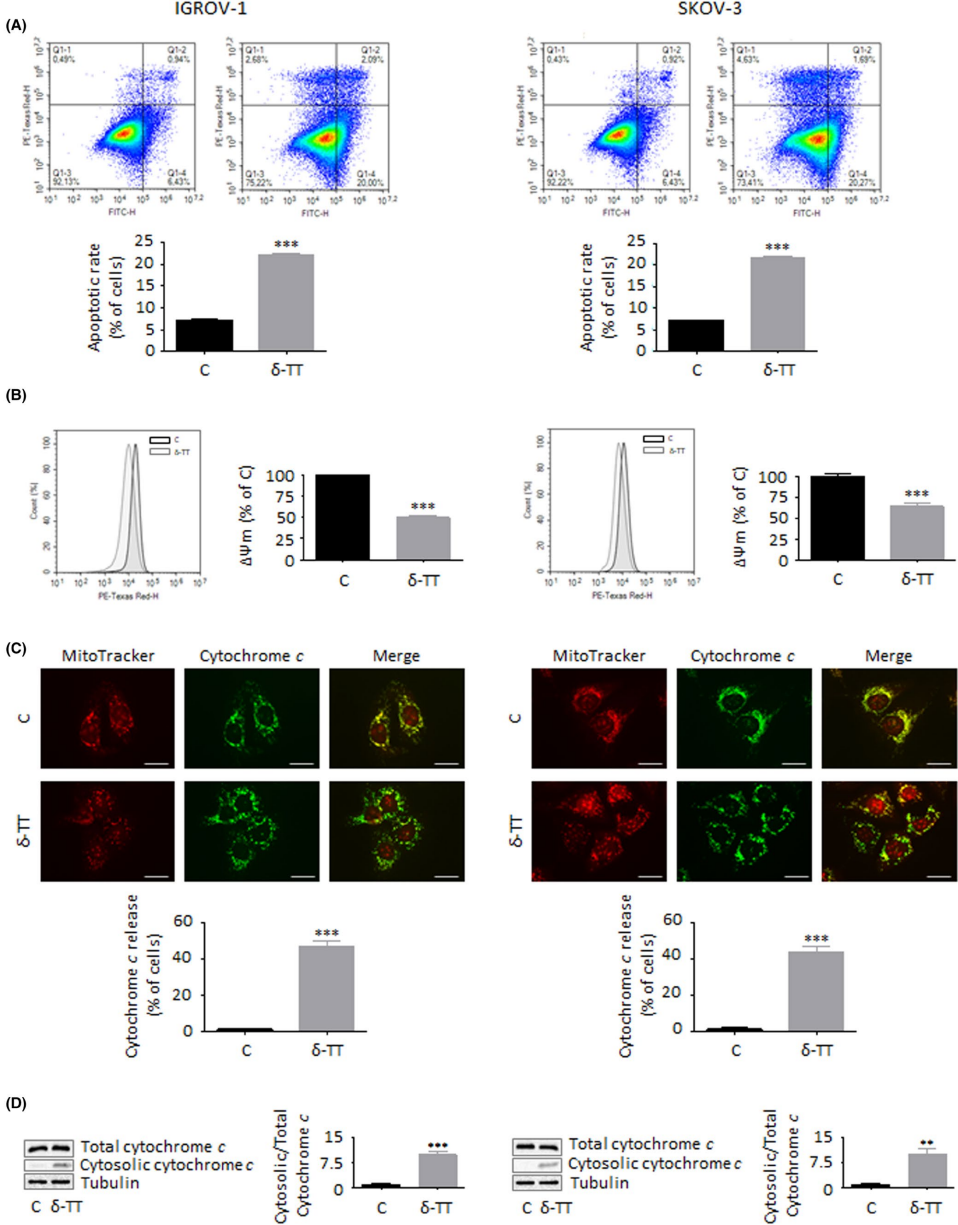
δ‐TT triggers mitochondrial apoptosis in OC cells. (A), IGROV‐1 and SKOV‐3 cells were treated with δ‐TT (15 μg/ml, 24 h); apoptotic rate was then evaluated by cytofluorimetric analysis after staining with eBioscience™ Annexin V‐FITC Apoptosis Detection Kit (according to the manufacturer's protocol). One representative of three experiments performed is shown. Data represent mean values ± SEM and were analysed by t test. ***P < .001 vs C, controls (vehicle). (B), Cells were treated with δ‐TT (15 μg/ml, 12 h); mitochondrial membrane potential (ΔΨm) was then evaluated by cytofluorimetric analysis after staining with the fluorescent dye MitoTracker Orange CMTMRos (10 µmol/L, 30 min). Each experiment was repeated three times. Data represent mean values ± SEM and were analysed by t test. ***P < .001 vs C, controls (vehicle). (C), Cells were treated with δ‐TT (15 μg/ml, 12 h); the intracellular localization of cytochrome *c* was then evaluated by immunofluorescence analysis. One representative of three experiments performed is shown. Scale bars are 20 μm. Data represent mean values ± SEM and were analysed by t test. ***P < .001 vs C, controls (vehicle). (D), Cells were treated with δ‐TT (15 μg/ml, 12 h); the intracellular localization of cytochrome *c* was then evaluated via Western blot analysis by using a Cell Fractionation Kit. One representative of three experiments performed is shown. Data represent mean values ± SEM and were analysed by t test. **P < .01 vs C, controls (vehicle); ***P < .001 vs C, controls (vehicle)

**FIGURE 4:**
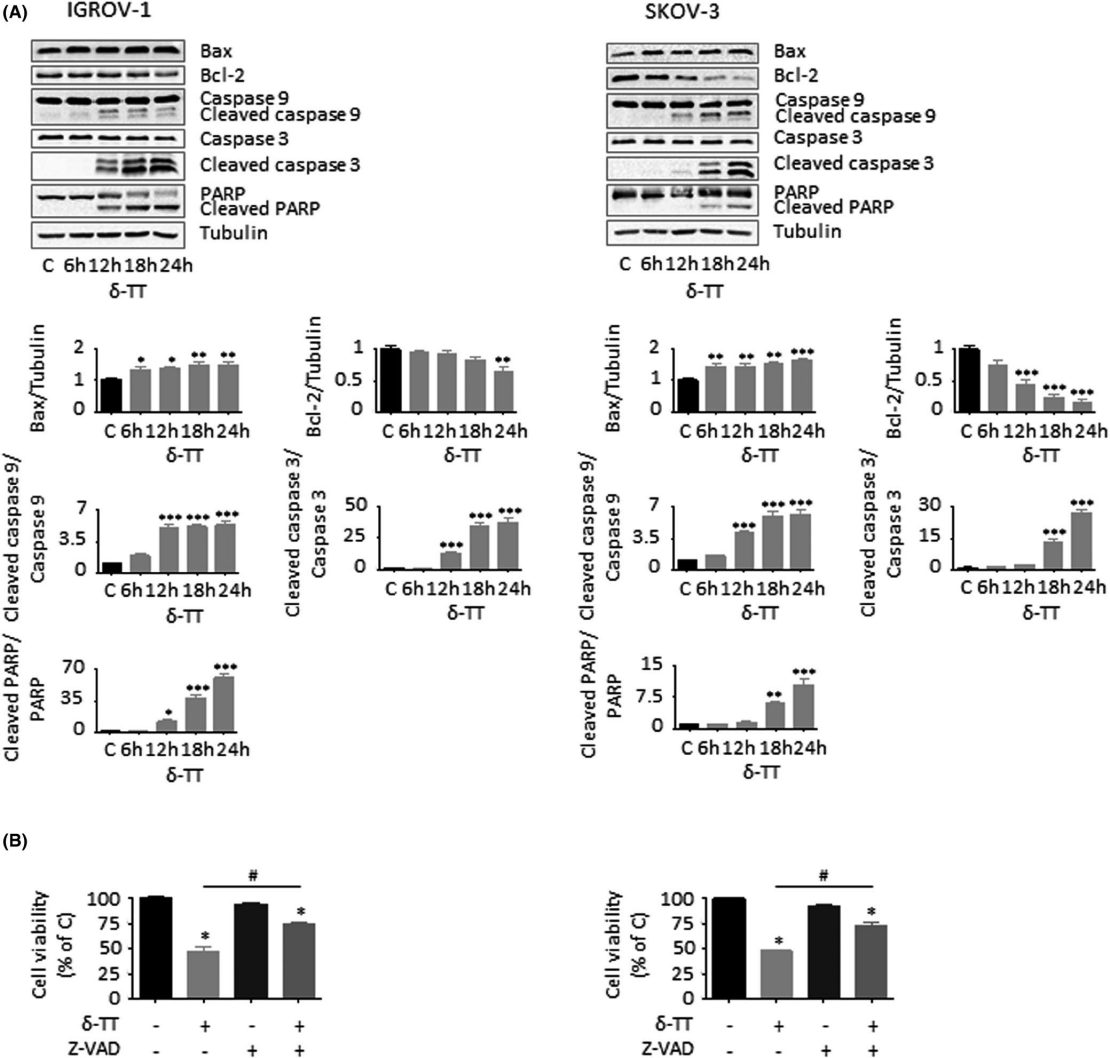
δ‐TT activates the intrinsic caspase cascade in OC cells. (A), After δ‐TT treatment (15 μg/ml, 1‐24 h), Western blot analysis was performed to investigate the expression levels of Bax, Bcl‐2, cleaved caspase 9, cleaved caspase 3 and PARP. Tubulin expression was evaluated as a loading control. One representative of three experiments performed is shown. Data represent mean values ± SEM and were analysed by Dunnett's test after one‐way analysis of variance. *P < .05 vs C, controls (vehicle); **P < .01 vs C, controls (vehicle); ***P < .001 vs C, controls (vehicle). (B), Cells were pretreated with the pan‐caspase inhibitor Z‐VAD‐FMK (50 μM, 4 h) and then with δ‐TT (15 μg/ml, 24 h). Cell viability was assessed by MTT assay. Each experiment was repeated three times. Data represent mean values ± SEM and were analysed by Bonferroni's test after one‐way analysis of variance. *P < .05 vs C, controls (vehicle). #*P* < .05 vs δ‐TT‐treated cells

Since inhibition of cell proliferation is strictly associated with apoptosis induction, we further investigated whether δ‐TT could trigger apoptotic OC cell death. Our data from Annexin V/PI staining indicate that the compound (15 μg/ml, 24 hours) induced apoptosis in both IGROV‐1 and SKOV‐3 cells, with apoptotic rates being around 22% in both cell lines (Figure 3A). Additionally, after 12 hours of treatment, both mitochondrial membrane potential (ΔΨm) loss and cytochrome *c* release were observed (Figure 3B,3C,3D), highlighting the activation of the apoptotic intrinsic pathway in OC cells; this hypothesis was also confirmed by the parallel upregulation of Bax/Bcl‐2 ratio and cleavage of caspase 9, caspase 3 and PARP (Figure 4A). Finally, pretreatment of both IGROV‐1 and SKOV‐3 cells with the pan‐caspase inhibitor Z‐VAD‐FMK (50 μmol/L, 4 hours) significantly counteracted δ‐TT cytotoxic effects (Figure 4B), demonstrating the involvement of mitochondrial apoptosis in the anti‐OC activity of the compound.

### 3.4 ROS generation is involved in the apoptosis induced by δ‐TT in OC cells

**FIGURE 5:**
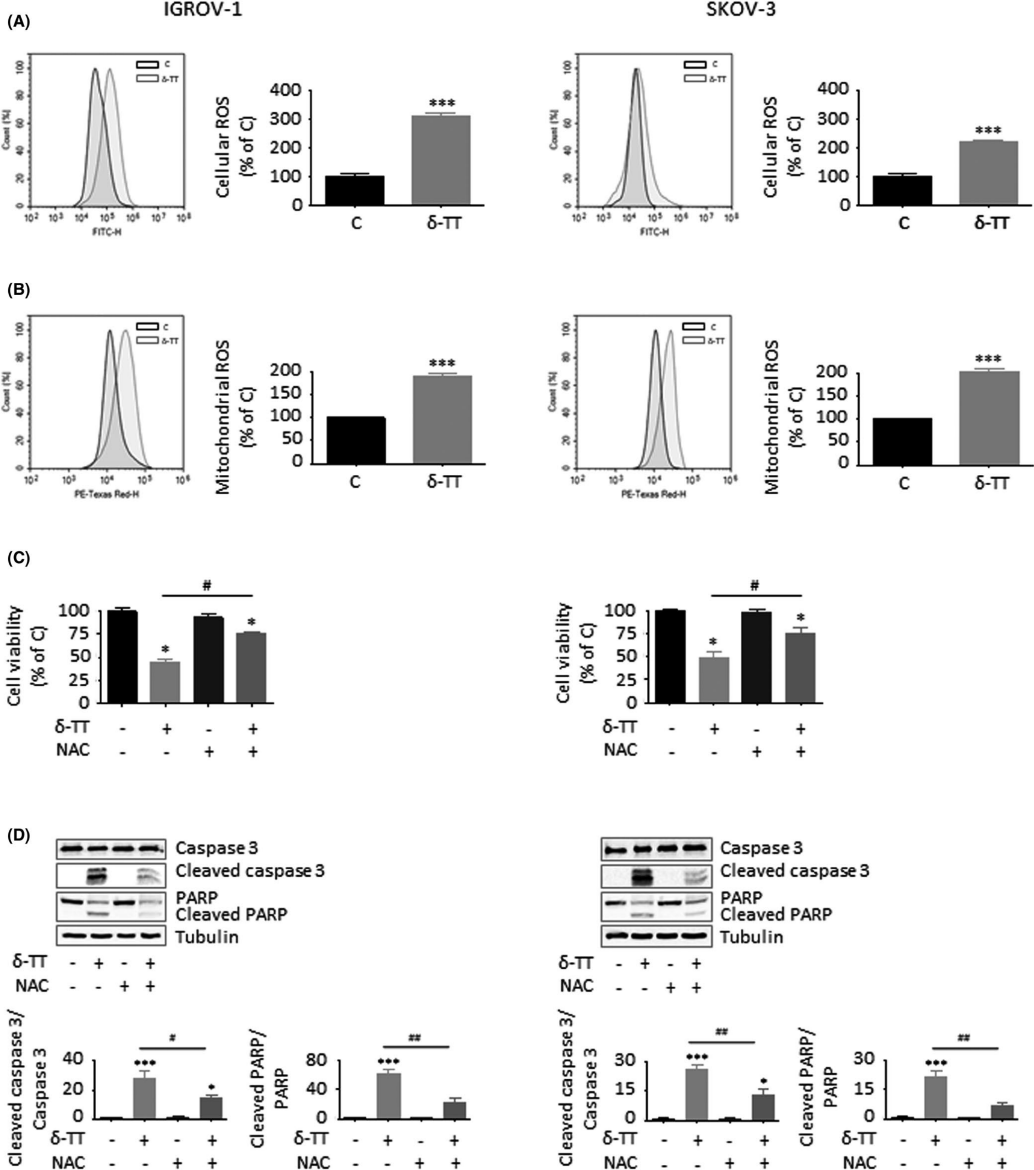
ROS generation is involved in the apoptosis induced by δ‐TT in OC cells. (A), IGROV‐1 and SKOV‐3 cells were treated with δ‐TT (15 μg/ml, 12 h); cellular ROS production was then evaluated by cytofluorimetric analysis after staining with 2',7'‐dichlorofluorescin diacetate (DCFDA, 10 µmol/L, 30 min). Each experiment was repeated three times. Data represent mean values ± SEM and were analysed by t test. ***P < .001 vs C, controls (vehicle). (B), Cells were treated with δ‐TT (15 μg/ml, 12 h); mitochondrial ROS production was then evaluated by cytofluorimetric analysis after staining with MitoSOX Red (5 μmol/L, 10 min). Each experiment was repeated three times. Data represent mean values ± SEM and were analysed by t test. ***P < .001 vs C, controls (vehicle). (C), Cells were pretreated with the ROS scavenger N‐acetyl‐L‐cysteine (NAC, 4 mmol/L, 2 h) and then with δ‐TT (15 μg/ml, 24 h). Cell viability was assessed by MTT assay. Each experiment was repeated three times. Data represent mean values ± SEM and were analysed by Bonferroni's test after one‐way analysis of variance. *P < .05 vs C, controls (vehicle). #*P* < .05 vs δ‐TT‐treated cells. (D), Cells were pretreated with NAC (4 mmol/L, 2 h) and then with δ‐TT (15 μg/ml, 24 h). Caspase 3 and PARP cleavage was evaluated by Western blot analysis. Tubulin expression was evaluated as a loading control. One representative of three experiments performed is shown. Data represent mean values ± SEM and were analysed by Bonferroni's test after one‐way analysis of variance. *P < .05 vs C, controls (vehicle); ***P < .001 vs C, controls (vehicle). #*P* < .05 vs δ‐TT‐treated cells; ##*P* < .01 vs C, controls (vehicle)

We have previously reported that δ‐TT is able to generate massive oxidative stress in prostate cancer and melanoma cells.^10^, ^11^, ^12^ Therefore, we measured ROS formation in both IGROV‐1 and SKOV‐3 cells treated with the compound. As described in Figure 5A,B, after 12 hours of treatment (15 μg/ml) OC cells exhibited a threefold (IGROV‐1) and twofold (SKOV‐3) increase in overall cellular ROS production and a twofold (both cell lines) increase in mitochondrial ROS generation as compared to control group. In particular, we demonstrated that pretreatment of tumour cells with NAC (4 mmol/L, 2 hours), a well‐known ROS scavenger, significantly counteracted δ‐TT (15 μg/ml, 24 hours) cytotoxicity (Figure 5C) and proapoptotic activity (Figure 5D), indicating that oxidative stress induction is crucially linked to the apoptosis triggered by the compound in OC cell lines.

### 3.5 MAPK activation is implicated in the ROS‐related apoptotic OC cell death caused by δ‐TT

**FIGURE 6:**
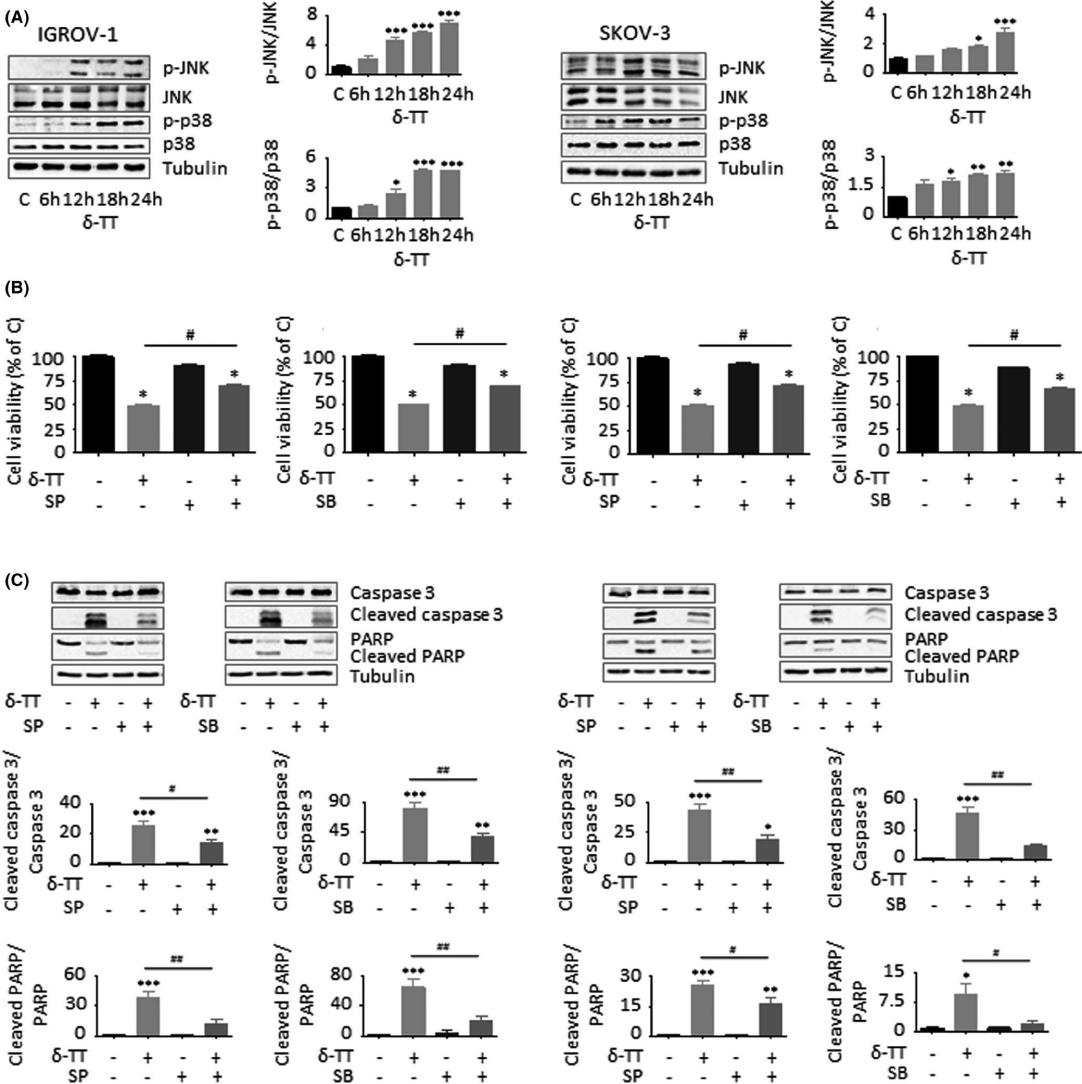
MAPK activation is implicated in the ROS‐related apoptotic OC cell death caused by δ‐TT. (A), After δ‐TT treatment (15 μg/ml, 1‐24 h), Western blot analysis was performed to investigate the expression levels of p‐JNK and p‐p38. Tubulin expression was evaluated as a loading control. One representative of three experiments performed is shown. Data represent mean values ± SEM and were analysed by Dunnett's test after one‐way analysis of variance. *P < .05 vs C, controls (vehicle); **P < .01 vs C, controls (vehicle); ***P < .001 vs C, controls (vehicle). (B), Cells were pretreated with JNK (SP600125, 20 μmol/L, 2 h) or p38 (SB203580, 20 μmol/L, 2 h) inhibitors and then with δ‐TT (15 μg/ml, 24 h). Cell viability was assessed by MTT assay. Each experiment was repeated three times. Data represent mean values ± SEM and were analysed by Bonferroni's test after one‐way analysis of variance. *P < .05 vs C, controls (vehicle). #*P* < .05 vs δ‐TT‐treated cells. (C), Cells were pretreated with SP600125 (20 μmol/L, 2 h) or SB203580 (20 μmol/L, 2 h) and then with δ‐TT (15 μg/ml, 24 h). Caspase 3 and PARP cleavage was evaluated by Western blot analysis. Tubulin expression was evaluated as a loading control. One representative of three experiments performed is shown. Data represent mean values ± SEM and were analysed by Bonferroni's test after one‐way analysis of variance. *P < .05 vs C, controls (vehicle); **P < .01 vs C, controls (vehicle); ***P < .001 vs C, controls (vehicle). #*P* < .05 vs δ‐TT‐treated cells; ##*P* < .01 vs C, controls (vehicle)

**FIGURE 7:**
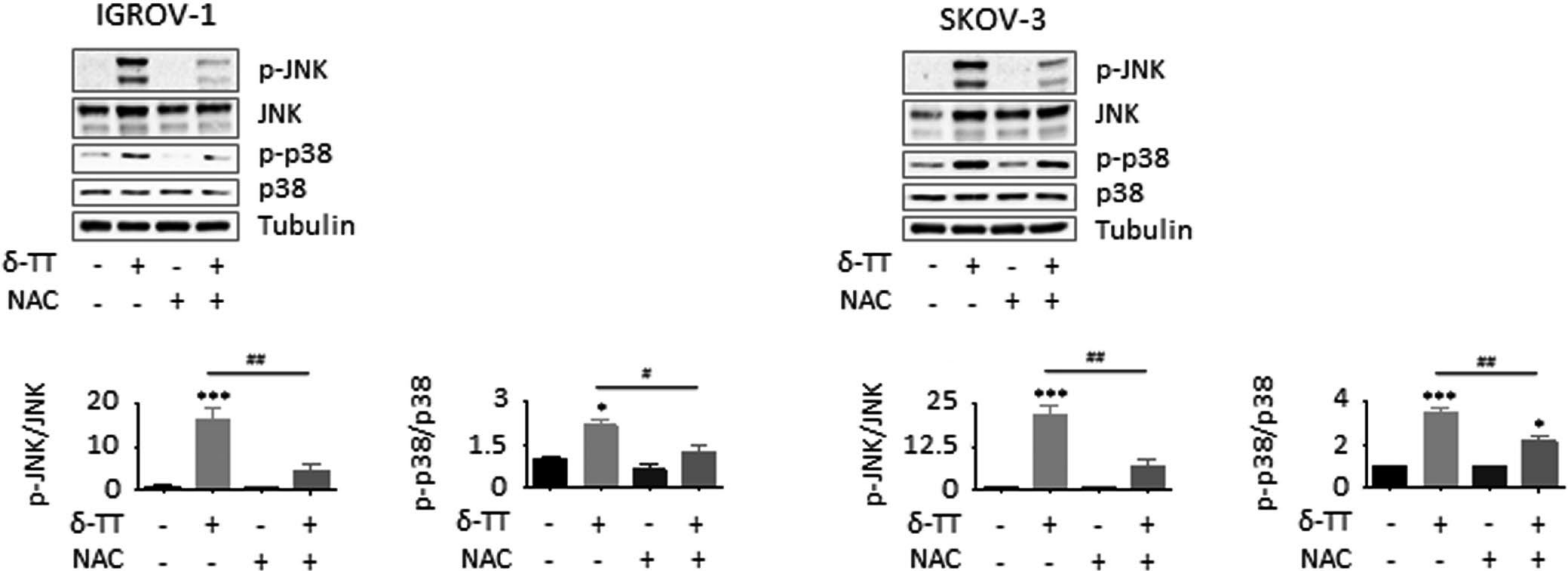
The ROS/MAPK axis mediates the apoptotic OC cell death triggered by δ‐TT. Cells were pretreated with NAC (4 mmol/L, 2 h) and then with δ‐TT (15 μg/ml, 24 h). JNK and p38 phosphorylation was evaluated by Western blot analysis. Tubulin expression was evaluated as a loading control. One representative of three experiments performed is shown. Data represent mean values ± SEM and were analysed by Bonferroni's test after one‐way analysis of variance. *P < .05 vs C, controls (vehicle); ***P < .001 vs C, controls (vehicle). #*P* < .05 vs δ‐TT‐treated cells; ##*P* < .01 vs C, controls (vehicle)

It is now well established that oxidative stress can mediate the activation of growth‐suppressing MAPK pathways able to promote Bax phosphorylation and translocation to mitochondria and to parallelly attenuate Bcl‐2 expression, eventually culminating in the initiation of the caspase cascade.^22^, ^23^, ^24^, ^25^, ^26^, ^27^ Thus, we analysed MAPK levels in both IGROV‐1 and SKOV‐3 cells treated with δ‐TT. As shown in Figure 6A, the compound (15 μg/ml, 24 hours) induced a time‐dependent phosphorylation of JNK and p38 proteins, which were found to modulate both δ‐TT cytotoxicity and proapoptotic activity; in fact, both JNK (SP600125, 20 μmol/L, 2 hours) and p38 (SB203580, 20 μmol/L, 2 hours) inhibitors successfully rescued OC cell viability (Figure 6B) and prevented caspase 3 and PARP activation (Figure 6C). In addition, pretreatment of tumour cells with NAC (4 mmol/L, 2 hours) markedly blocked δ‐TT‐related MAPK activation (15 μg/ml, 24 hours), suggesting that JNK and p38 upregulation is critically implicated in the ROS‐mediated apoptotic OC cell death caused by the compound (Figure 7).

### 3.6 δ‐TT synergizes with cisplatin in reducing OC cell viability

**FIGURE 8:**
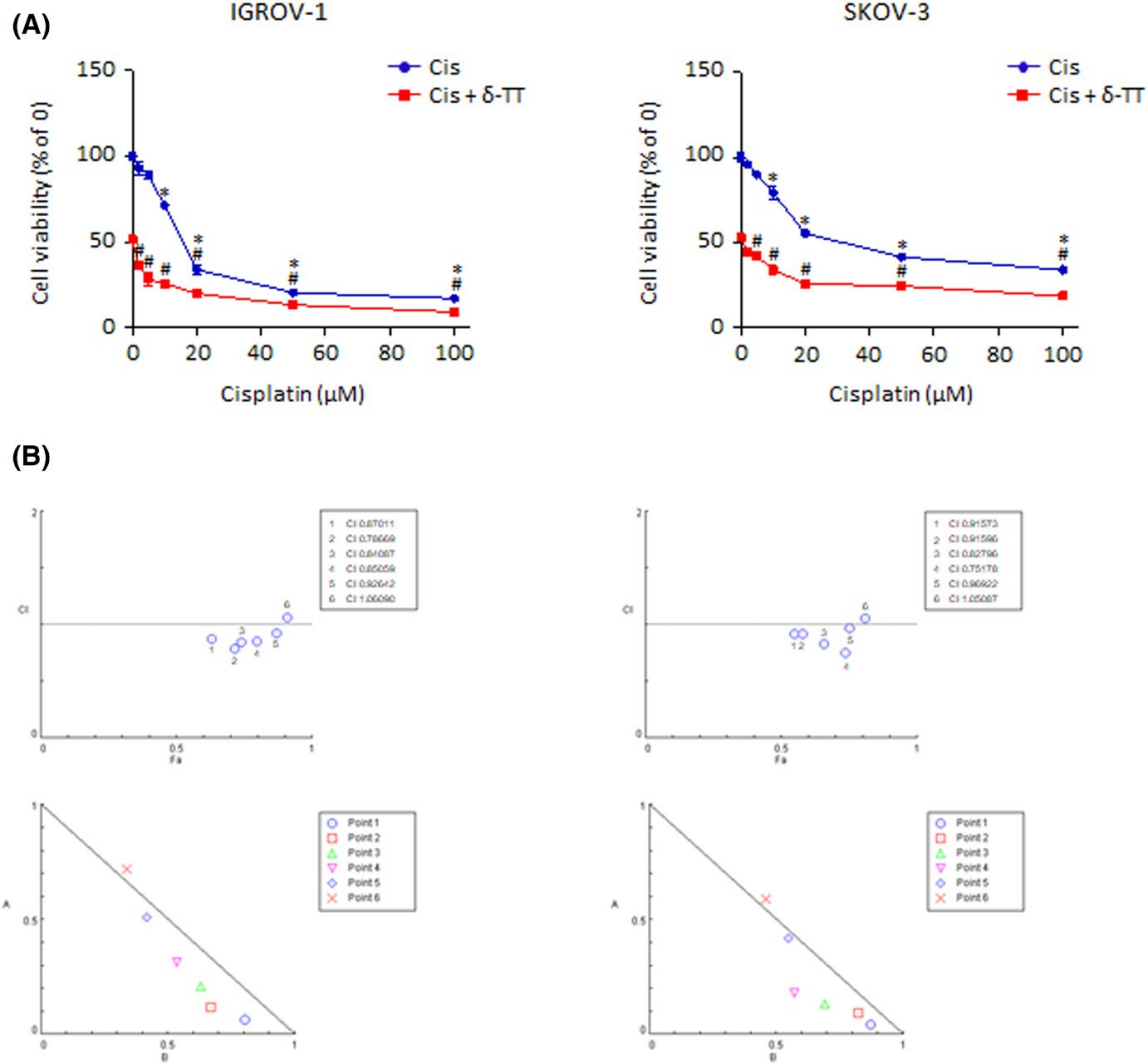
δ‐TT synergizes with cisplatin in reducing OC cell viability. (A), IGROV‐1 and SKOV‐3 cells were treated with cisplatin (2‐100 μmol/L), alone or in combination with δ‐TT (15 μg/ml) for 24 h. Cell viability was then evaluated by MTT assay. Each experiment was repeated three times. Data represent mean values ± SEM and were analysed by Dunnett's test after one‐way analysis of variance. *P < .05 vs C, controls (vehicle). #*P* < .05 vs δ‐TT‐treated cells. (B), Data from Figure 6A were converted to Fraction Affected (FA) and plotted against Combination Index (CI). Straight line on the graph designates a CI equal to 1. Combination Index interpretation was as follows: CI value of 1 indicates additivity; CI<1 indicates synergism; and CI >1 indicates antagonism

To investigate whether δ‐TT could enhance cisplatin cytotoxicity in OC cell lines, we treated both IGROV‐1 (platinum‐sensitive) and SKOV‐3 (platinum‐resistant) cells with various concentrations of the latter (2, 5, 10, 20, 50, 100 μmol/L), alone or in combination with the natural compound (15 μg/ml) (Figure 8A). After 24 hours, cisplatin IC50 value was found to be of 19.75 μmol/L for IGROV‐1 cells and of 38.87 μmol/L for SKOV‐3 cells, which decreased significantly to 0.71 μmol/L and 1.30 μmol/L upon combination with δ‐TT. Notably, these observations were also confirmed by isobologram analysis (Figure 8B), which evidenced the synergism between the two anti‐tumour agents in promoting OC cell death.

**FIGURE 9:**
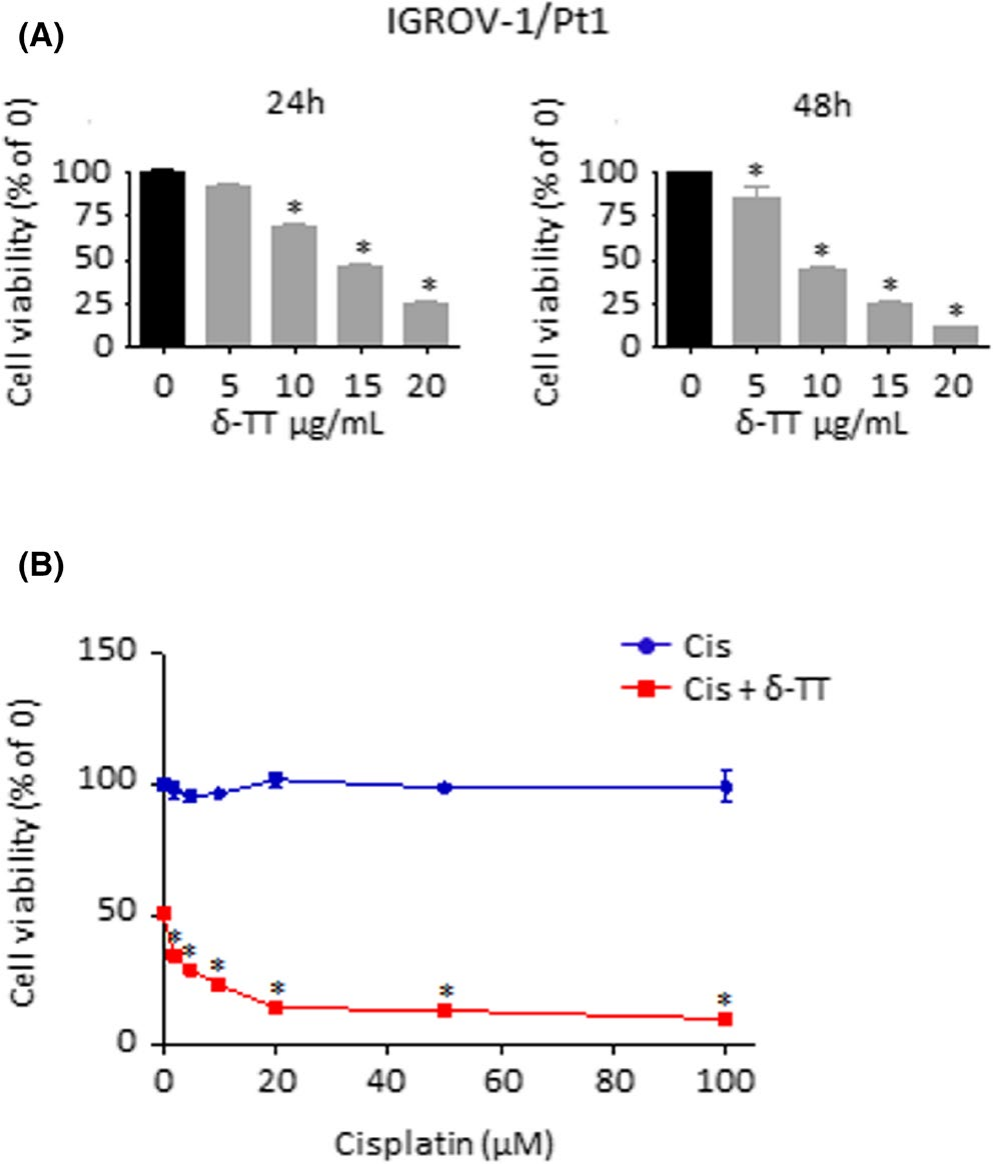
δ‐TT mediates cytotoxic effects on IGROV‐1/Pt1 cells and re‐sensitizes them to chemotherapy. (A), IGROV‐1/Pt1 cells were treated with δ‐TT (5‐20 μg/ml) for 24 and 48 h. Cell viability was then evaluated by MTT assay. Each experiment was repeated three times. Data represent mean values ± SEM and were analysed by Dunnett's test after one‐way analysis of variance. *P < .05 vs C, controls (vehicle). (B), IGROV‐1/Pt1 cells were treated with cisplatin (2‐100 μmol/L), alone or in combination with δ‐TT (15 μg/ml) for 24 h. Cell viability was then evaluated by MTT assay. Each experiment was repeated three times. Data represent mean values ± SEM and were analysed by Dunnett's test after one‐way analysis of variance. *P < .05 vs C, controls (vehicle)

To further validate the above results, we tested δ‐TT efficacy on chemotherapy‐adapted IGROV‐1/Pt1 cells, in the absence or presence of cisplatin. As shown in Figure 9A,B, the compound was able not only to mediate concentration‐dependent cytotoxic effects when given alone (5‐20 μg/ml, 24‐48 hours, IC50 value of 3.41 × 10^−5^ M) but also to re‐sensitize the chemoresistant OC cell line to platinum treatment (δ‐TT 15 μg/ml and cisplatin 2, 5, 10, 20, 50, 100 μmol/L, 24 hours), demonstrating great potential in both mono‐ and combination therapy.

## 4 DISCUSSION

It is now widely accepted that TTs possess potent anti‐tumour properties.^8^, ^9^ Although most of the studies so far reported were performed with γ‐TT, the δ isomer has recently shown clinical promise in the treatment of recurrent OC when given in combination with bevacizumab.^13^ However, its mechanism of action is still poorly understood.

Here, we dissected the molecular mechanisms underlying the anti‐tumour activity of δ‐TT in two different OC cell lines, IGROV‐1 and SKOV‐3 cells, known to be cisplatin‐sensitive and ‐resistant, respectively.

We found that δ‐TT can exert significant anti‐tumour effects on OC cells, by decreasing both cell viability and proliferation. Specifically, we demonstrated that the compound can trigger G1 phase cell cycle arrest, accompanied by cyclin D1, cyclin D3, CDK4 and CDK6 downregulation and by p21 and p27 activation, and intrinsic apoptosis, paralleled by mitochondrial membrane potential loss, cytochrome *c* release, Bax/Bcl‐2 ratio upregulation and caspase 9, caspase 3 and PARP cleavage. In particular, the involvement of the intrinsic apoptotic pathway in the anti‐OC activity of δ‐TT was confirmed by the Z‐VAD‐FMK‐mediated suppression of the compound cytotoxicity. These data are consistent with previous reports describing the antiproliferative and proapoptotic effects elicited by TTs in different malignancies, including breast, colon, gastric, lung, pancreatic, prostate, skin and liver cancer.^8^, ^9^

To get further insights into the molecular targets of δ‐TT, we focused our attention on the analysis of intracellular oxidative stress. Indeed, several natural compounds have been shown to induce ROS‐related cell death in different types of tumour, including OC.^28^, ^29^, ^30^, ^31^ Regarding TTs, the γ isoform has been reported to trigger mitochondrial dysfunction‐ and oxidative imbalance‐associated apoptosis in gastric adenocarcinoma cells, as well as to sensitize colorectal cancer cells to TRAIL proapoptotic activity via ROS overproduction.^32^, ^33^ Similarly, δ‐TT can cause ROS‐mediated cytotoxicity in various models of breast and prostate cancer,^11^, ^34^ while α‐tocopheryl succinate, a redox‐silent Vitamin E analogue, has been demonstrated to stimulate cytochrome *c* release in neuroblastoma through a severe alteration of redox homeostasis.^35^ In OC cells, we observed that δ‐TT promotes mitochondrial ROS generation and that NAC can successfully rescue cell viability by preventing caspase 3 and PARP cleavage, thus highlighting the involvement of oxidative stress in the apoptosis induced by the compound.

Since ROS generation is often linked to MAPK activation,^22^ we analysed p‐JNK and p‐p38 expression in δ‐TT‐treated OC cells. A significant increase in the levels of both these proteins was found, and it was shown to be significantly counteracted by NAC‐mediated ROS scavenging. Interestingly, by using SP600125 and SB203580, upregulation of both p38 and JNK was demonstrated to correlate with δ‐TT‐related cytotoxicity and apoptosis. Notably, numerous phytochemicals have been shown to specifically target the MAPK signalling in a variety of cancers.^36^, ^37^ In particular, TTs are known to trigger JNK‐ and p38‐dependent cell death in several types of tumour, such as melanoma, lymphoma and breast and prostate cancer.^10^, ^11^, ^38^, ^39^, ^40^ In this setting, our data not only support previous findings about the MAPK‐targeting ability of TTs but also indicate that the induction of a ROS/MAPK cascade is deeply implicated in the anti‐OC effects of δ‐TT.

Platinum‐based chemotherapy represents the standard treatment for OC.^4^ However, it is generally associated with high toxicity and relapse rates.^5^, ^6^, ^7^ In order to overcome these issues, we conducted a combination study with δ‐TT, evidencing a strong synergistic in vitro interaction between the two anticancer agents not only in platinum‐sensitive (IGROV‐1) OC cells but also in two models of drug resistance (wild type SKOV‐3 and chemotherapy‐adapted IGROV‐1/Pt1 cell lines). Remarkably, several natural compounds are able to sensitize cancer cells to cisplatin^41^; among them, berberine, β‐elemene, genistein, hirsutenone, morin, saikosaponin‐d, cardamonin, theaflavin‐3,3'‐digallate and withaferin A have been found to specifically enhance the chemosensitivity of OC cell lines.^42^, ^43^, ^44^, ^45^, ^46^, ^47^, ^48^, ^49^, ^50^ Furthermore, δ‐TT itself has been reported to synergize with a wide range of anti‐tumour drugs, including common anti‐OC chemotherapeutics (i.e. taxanes)^8^, ^9^; in particular, its ability to overcome chemoresistance in tumours seems to be associated with the induction of multiple growth‐suppressive and pro‐death pathways, as well as with a specific targeting of the mechanisms responsible for treatment escape, such as growth factor receptor hyperexpression, ATP‐binding cassette (ABC) transporter activation and cancer stem cell (CSC) propagation.^8^, ^9^, ^51^ To our knowledge, this is the first study highlighting the synergism between δ‐TT and cisplatin in OC.

In conclusion, these results demonstrate that δ‐TT exerts a potent anti‐tumour activity in OC cell lines by triggering both G1 phase cell cycle arrest and mitochondrial apoptosis. In particular, we showed that it can induce MAPK/ROS‐mediated OC cell death and enhance cisplatin anti‐OC efficacy, providing a deeper understanding of its anti‐tumour properties.

## CONFLICT OF INTEREST

The authors declare no conflict of interest.

## AUTHOR CONTRIBUTIONS

FF designed the study. FF, MM, MR, VZ and NZ collected and analysed the data. FF, MM, MR, VZ, NZ and PL prepared the manuscript. PL was involved in funding acquisition.

## Data Availability

The data that support the findings of this study are available from the corresponding author upon reasonable request.
